# Differential expression spectrum and targeted gene prediction of tRNA-derived small RNAs in idiopathic pulmonary arterial hypertension

**DOI:** 10.3389/fmolb.2023.1204740

**Published:** 2023-07-11

**Authors:** Yusi Chen, Yi Tang, Sitong Hou, Jun Luo, Jingyuan Chen, Haihua Qiu, Wenjie Chen, Kexing Li, Jin He, Jiang Li

**Affiliations:** ^1^ Department of Cardiovascular Medicine, The Second Xiangya Hospital of Central South University, Changsha, Hunan, China; ^2^ Clinical Medicine Research Center of Heart Failure of Hunan Province, Department of Cardiology, Hunan Provincial People’s Hospital, The First Affiliated Hospital of Hunan Normal University, Hunan Normal University, Changsha, China; ^3^ Clinical Medicine, Xiangya Medical School of Central South University, Changsha, Hunan, China; ^4^ Department of Pharmacology, Hebei University, Baoding, Hebei, China

**Keywords:** pulmonary arterial hypertension, tRNA-derived small RNAs, non-coding RNAs, small RNA microarray, bioinformatics

## Abstract

**Background:** Idiopathic pulmonary arterial hypertension (PAH) is a potentially fatal pulmonary vascular disease with an extremely poor natural course. The limitations of current treatment and the unclear etiology and pathogenesis of idiopathic PAH require new targets and avenues of exploration involved in the pathogenesis of PAH. tRNA-derived small RNAs (tsRNAs), a new type of small non-coding RNAs, have a significant part in the progress of diverse diseases. However, the potential functions behind tsRNAs in idiopathic PAH remain unknown.

**Methods:** Small RNA microarray was implemented on three pairs of plasma of idiopathic PAH patients and healthy controls to investigate and compare tsRNAs expression profiles. Validation samples were used for real-time polymerase chain reaction (Real-time PCR) to verify several dysregulated tsRNAs. Bioinformatic analysis was adopted to determine potential target genes and mechanisms of the validated tsRNAs in PAH.

**Results:** Microarray detected 816 statistically significantly dysregulated tsRNAs, of which 243 tsRNAs were upregulated and 573 were downregulated in PAH. Eight validated tsRNAs in the results of Real-time PCR were concordant with the small RNA microarray: four upregulated (tRF3a-AspGTC-9, 5’tiRNA-31-GluCTC-16, i-tRF-31:54-Val-CAC-1 and tRF3b-TyrGTA-4) and four downregulated (5’tiRNA-33-LysTTT-4, i-tRF-8:32-Val-AAC-2, i-tRF-2:30-His-GTG-1, and i-tRF-15:31-Lys-CTT-1). The Gene Ontology analysis has shown that the verified tsRNAs are related to cellular macromolecule metabolic process, regulation of cellular process, and regulation of cellular metabolic process. It is disclosed that potential target genes of verified tsRNAs are widely involved in PAH pathways by Kyoto Encyclopedia of Genes and Genomes.

**Conclusion:** This study investigated tsRNA profiles in idiopathic PAH and found that the dysregulated tsRNAs may become a novel type of biomarkers and possible targets for PAH.

## Background

Idiopathic pulmonary arterial hypertension (PAH) is a potentially fatal pulmonary vessel disorder based on progressive pulmonary vascular remodeling resulting in the obliteration of distal pulmonary arteries and increased pulmonary vascular resistance (Ruopp and Cockrill, 2022). PAH, especially idiopathic PAH has been regarded as a cancer-like disease owing to its pathological mechanism exhibiting dysregulated cellular metabolism, sustained proliferation, and escape from apoptosis (Wu et al., 2021). The natural course of idiopathic PAH involves extremely poor outcomes, with a mean survival of 2–3 years from diagnosis if left untreated and a survival rate of 34% at 5 years (D'Alonzo et al., 1991) (Farber et al., 2015). Although PAH-targeted medications have benefited PAH patients a lot (Zhang et al., 2011), the current long-term prognosis for PAH patients remains terrible and PAH is incompletely curable. Thus, the limitations of current treatment and the unclear etiology and pathogenesis of PAH require new targets and avenues of exploration involved in the pathogenesis of PAH.

With the rapid advancement of small RNA microarray technology, new non-coding RNAs are being discovered continuously. Non-coding RNAs as a PAH regulatory target have good specificity and sensitivity (Tang, 2020). Researches suggest that long non-coding RNA H19 is involved in right ventricular failure in PAH (Omura et al., 2020). MicroRNAs (miRNAs) participate in the onset and development of PAH and have been a hot spot for PAH (Zhang et al., 2020). tRNA-derived small RNAs (tsRNA) are structurally and functionally similar to miRNAs (Green et al., 2020); however, they are superior to the other non-coding RNAs in terms of stability and abundance (Selitsky et al., 2015).

tsRNAs are precisely cleaved into regulatory-functional, exclusive-sized fragments in the context of stress via specified nucleases (such as dicer and angiogenin) from mature or precursor tRNA (Pereira et al., 2021). Generally, there exist two types of tsRNAs depending on the length and cleavage sites: tRNA-derived fragments of 14∼30 nucleotides in length and tRNA-derived stress-induced RNAs generated by specific cuts in the anticodon loop of 28–36 nucleotides (Su et al., 2019). Through the widespread development of high throughput microarray, growing evidence suggested that tsRNAs are not just random cleavage of tRNA, but a small non-coding RNA with a key role in regulating pathophysiological mechanisms, such as cell proliferation, apoptosis, and migration (Xie et al., 2020). The abnormally expressed tsRNAs are reportedly relevant to pancreatic cancer and metabolic disorders (Chen et al., 2016; Jin et al., 2021). Likewise, idiopathic PAH patients usually have accompanying metabolic disorders and idiopathic PAH behaves as a carcinoid phenomenon (Li et al., 2021; Wu et al., 2021). To the best of our knowledge, the research between tsRNAs and PAH has been blank, and their possible molecular mechanism in the pathophysiology governing PAH remains unknown. Therefore, we postulated that dysregulated tsRNAs may target some key mRNAs through different pathways involved in PAH. Our study is the first to perform tsRNA sequencing of idiopathic PAH to screen and verify PAH-specific biomarkers. Furthermore, we used bioinformatics methods to map the network of validated tsRNA interactions and predicted the potential role of target genes for dysregulated tsRNAs in order to identify novel targets in PAH (Benincasa et al., 2023a; Benincasa et al., 2023b). Lastly, we explored the potential molecular pathways of tsRNAs in PAH. These findings may offer distinctive perspectives in the search for potential molecular targets and new biomarkers for PAH in clinical practice.

## Materials and methods

### Clinical specimens

The study was approved by the Ethics Committee of the Second Xiangya Hospital of Central South University. All subjects signed an informed consent protocol. Between September 2019 and September 2021, 3 adult patients initially diagnosed with idiopathic PAH according to the criteria proposed by the European Society of Cardiology/European Respiratory Society guidelines in 2015 were enrolled (Galiè et al., 2016), and subject to a small RNA microarray comparison with three age-and sex-matched healthy controls. Then, a total of six unmatched specimens were used to validate the tsRNA expression by real-time polymerase chain reaction (Real-time PCR). The study flowchart is displayed in Supplementary Figure S1. All PAH patients underwent right heart catheterization. To avoid possible perturbation of the outcomes of the assay, patients diagnosed with other groups of pulmonary hypertension, accompanied with other cardiovascular diseases or metabolic diseases, and taking PAH-targeted medications, such as prostacyclin and its analogs, were eliminated from the study. The blood samples from peripheral veins immediately were reserved at −80°C after being purified until further analysis.

### RNA extraction and small RNA microarray

Total RNA was isolated from six plasma specimens using TRIzol (Invitrogen) based on the manufacturer’s protocol. Then, RNA quantity was measured with a NanoDrop ND-1000 spectrophotometer, and RNA integrality was evaluated by denaturing agarose gel electrophoresis. RNA samples were preconditioned using the rtStar tsRNA Pretreatment kit (Cat# AS-FS-005, Arraystar) to strip RNA of modifications such as acetylation and methylation that would interfere with small RNA microarray library construction. 100 ng total RNA was first dephosphorylated to remove both (P) and (cP) chemical groups from the 3′ end of RNAs to form a 3-OH end. Arraystar Small RNA Microarray contains more than 14,000 probes to specifically quantify tsRNAs. The labeled sample mixtures were hybridized on a microarray. Agilent G2505C microarray scanner scanned the slides.

### tsRNA data analysis

The scanned microarray images were imported into Agilent Feature Extraction software (version 11.0.1.1) for raw intensity data extraction. To compare idiopathic PAH patients and healthy controls for differential tsRNA expression, the fold change (FC) and statistical significance of the difference (*p*-value) were calculated for each small RNA. The log intensity and log2 order normalization were performed for the original data. After normalization, at least three samples of the six samples were held with probe signals having Present (P) or Marginal (M) QC flags. Then the probe signals were grouped and analyzed accordingly following signal QC flag filtering. Differentially expressed small RNAs between PAH patients and healthy controls were identified by filtering at the indicated fold change >2.0 and *p* < 0.05 was regarded as a significant difference. R software is used to draw hierarchical clustering, scatter plots, and volcano plots.

### Validation using real-time PCR

To verify the results of small RNA microarrays, eight tsRNAs were selected and their expression were verified in three PAH patients and three healthy controls. Total RNA was extracted from plasma according to the method described above. RNA was then converted to cDNA by reverse transcription as the producer’s protocol using the rtStar First-Strand cDNA Synthesis kit (Cat# AS-FS-003, Arraystar). Real-time PCR was carried out by QuantStudio 5 Real-time PCR System (Applied Biosystems), using selected primers (Supplementary Table S1) and 2X PCR master mixArraystarAS-MR-006-5. Thermo cycling conditions: initial denaturation at 95°C for 10 min; thereafter, 40 cycles were performed at 95°C for 10 s and 60°C for 60 s. U6 acted as an internal control and evaluated the efficiency of the reaction. Real-time PCR was done with three replicate wells for one sample. The 2^−△Cq^ method was used to analyze data. The amplification efficiency was determined using standard dilution curves.

### Bioinformatics analysis

To determine the potential target genes of the validated tsRNAs, TargetScan and miRanda were screened with the criteria of energy < −16, structure ≥140, and context < −0.18. After that, the predicted target genes were entered into a Gene Ontology (GO) database to analyze their molecular functions covering biological processes, cellular components, and molecular function. Significant pathways for these target genes were identified by Kyoto Encyclopedia of Genes and Genomes (KEGG) database. DAVID software was applied to depict the pathway diagram. Fisher’s exact test was used to analyze the results, with a *p*-value < 0.05 assumed meaningful.

### Statistical analysis

Data are expressed as mean ± standard deviation and were analyzed by R software version 4.0.2. Differences between groups were analyzed by Student’s *t*-test. Differences with a *p* < 0.05 were regarded as statistically significant.

## Results

### Altered expression spectrums of tsRNAs

**FIGURE 1:**
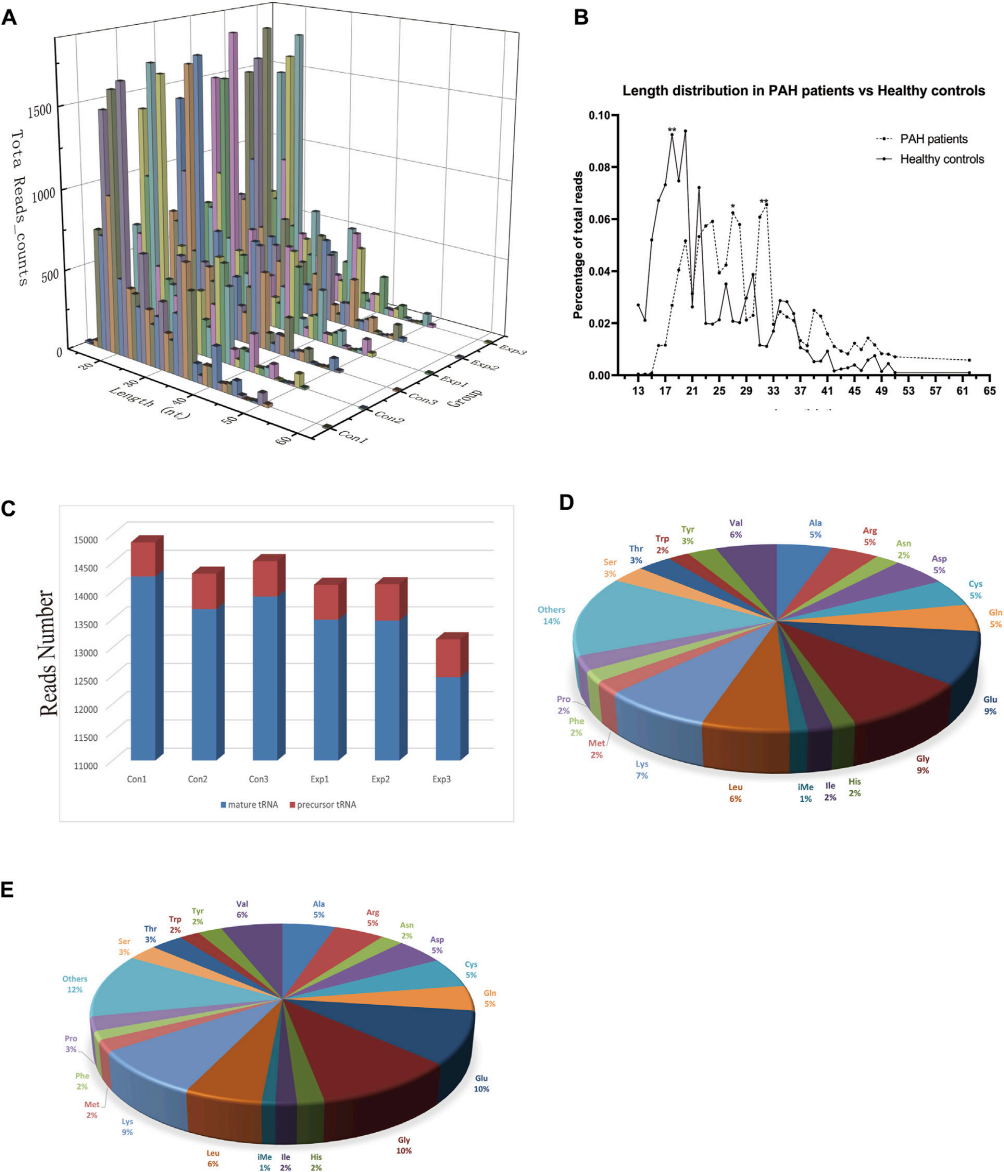
Characteristics of the detected tsRNA in PAH and healthy controls. **(A)** Length distribution of the detected tsRNAs. *x*-axis, the length of the detected tsRNAs; *y*-axis, the abundance of tsRNAs classified by different lengths. **(B)** The length of tsRNAs in PAH patients and matched healthy controls mainly ranged from 13 to 61 nt. **(C)** Reads number of the source of tsRNAs (mature tRNA and precursor tRNA). **(D,E)** Distribution of source tRNAs in PAH patients and healthy controls.

**FIGURE 2:**
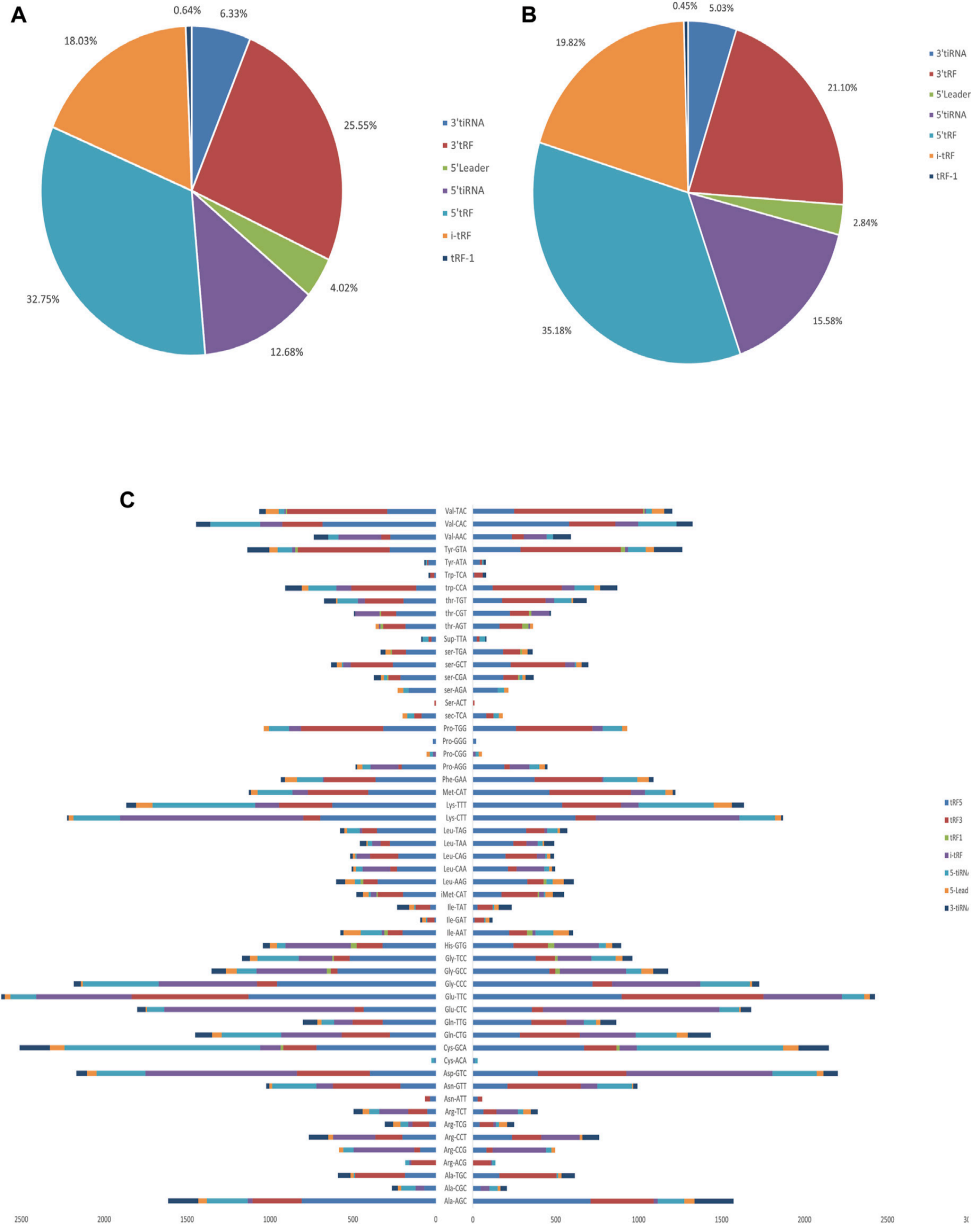
Proportions of subtype tsRNAs against tRNA iso-decoders between PAH patients and healthy controls. **(A)** Proportions of tsRNAs subtype in PAH patients. **(B)** Proportions of tsRNAs subtype in healthy controls. **(C)** Stacked plot for all subtypes of tsRNAs between PAH patients and healthy controls clustering by the anticodon of the tRNAs. The *X*-axis represents the number of all subtype tsRNAs derived from the same anticodon tRNA, and the *Y*-axis shows the tRNAs with the same anticodon.

The baseline characteristics of PAH patients and healthy controls are summarized in Table 1. Overall, the study included three PAH patients (1 female, 2 male) with 21–59 years, compared to three age and sex-matched healthy controls. According to the results of Supplementary Table S2, the extracted RNAs could be used for the following tsRNA experiments. For the first time, a small RNA microarray was used to identify tsRNAs expression profiles in PAH patients vs. healthy controls. Figure 1A depicted the distribution of tsRNA read length. Figure 1B demonstrated statistical differences between the two groups at 16, 27, and 32 nt. The number of tsRNAs derived from mature tRNAs was significantly lower in PAH patients than in the controls (Figure 1C). Among the source tRNAs, we observed that tRNAs-Gly and tRNAs-Glu were the most abundant, both accounting for 9% in the PAH group (Figure 1D) and both 10% in the healthy controls (Figure 1E). These tsRNAs from different tRNA precursors however may share the same anticodon. Figures 2A, B depicts the number of categories of tsRNAs between groups. In terms of numbers, the 5’tRF increased sharply, followed by 3’tRF in both groups. We found that Ser-ACT and Pro-GGG only had one type tsRNA in the two groups, while Cys-GCA, Gly-GCC, Gly-TCC, His-GTG, Ile-AAT, iMet-CAT, Tyr-GTA, and Val-TAC possessed all types of tsRNAs (Figure 2C).

**TABLE 1:** Clinical characteristics.

Participants no.	1	2	3	4	5	6
Group	Idiopathic PAH	Idiopathic PAH	Idiopathic PAH	Healthy control	Healthy control	Healthy control
Age (years)	28	59	21	25	48	26
Sex	female	male	male	female	male	male
WHO FC	3	3	3	NA	NA	NA
6MWD (meters)	397	367	412	526	543	513
mRAP (mmHg)	10	6	10	NA	NA	NA
mPAP (mmHg)	55	59	59	NA	NA	NA
PVR (Wood)	26.1	25.8	13.7	NA	NA	NA
CO (L/min)	1.8	2.0	3.8	5.3^a^	4.6^a^	3.9^a^

^a^ CO was obtained from echocardiography.

PAH, pulmonary arterial hypertension; NA, not applicable; WHO FC, World Health Organization functional class; 6MWD, 6-min walk distance; mRAP, mean right atrial pressure; mPAP, mean pulmonary arterial pressure; PVR, pulmonary vascular resistance; CO, cardiac output.

### Differentially expressed tsRNA in PAH

**FIGURE 3:**
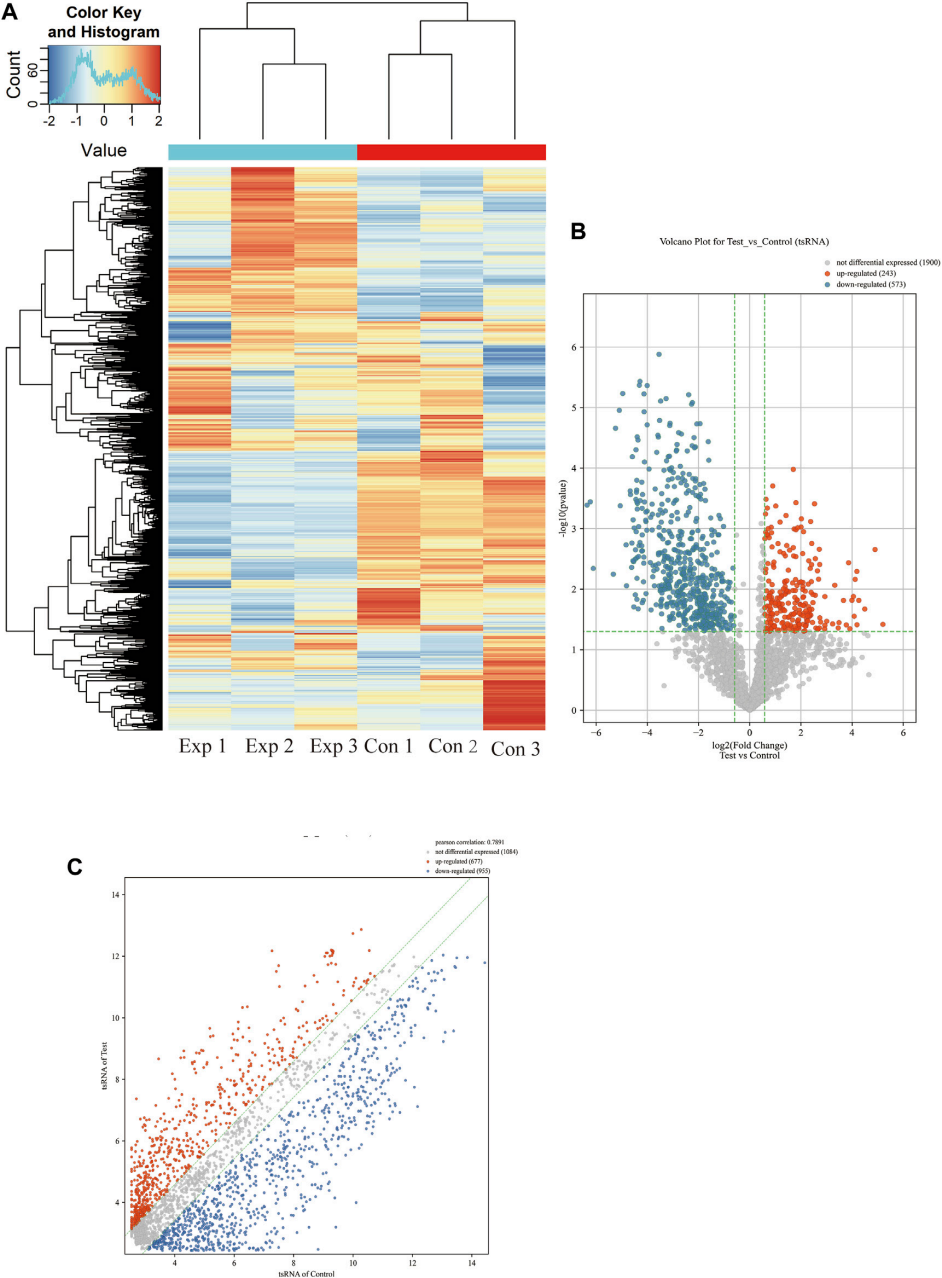
Expression profiles of all detected tsRNAs sequencing data in PAH patients and healthy controls. **(A)** Expression heatmap of all detected tsRNAs. **(B)** Volcano maps of differentially expressed tsRNAs in PAH patients (Test) vs. healthy controls. The vertical dotted lines manifest 2.0-fold changes (log2 scaled) up and down, respectively, and the horizontal dotted line shows a *p*-value of 0.05 (-log10 scaled). The red and blue points denote the significantly different expressed tsRNAs. **(C)** Scatter plots between the two groups. The values of the *X* and *Y*-axes in the scatter plot are the averaged transcript per million values of each group (log2 scaled). tsRNAs above the top line (red dots, upregulation) or below the bottom line (blue dots, downregulation) indicate more than 1.5-fold change between two compared groups. Gray dots indicate non-differentially expressed tsRNAs.

More than 2,700 tsRNA were detected from the three pairs of samples, of which 1,384 tsRNAs were not included by the MINTbase or tRFdb database. Hierarchical clustering was taken to visualize the tsRNAs expression spectrum in Figure 3A. At a folding variation greater than 1.5 and *p*-value < 0.05, tsRNAs are counted as significantly differential expressions. A series of 816 differential tsRNAs were visualized by volcano plots, and more downregulated tsRNAs (*n* = 573) were observed than upregulated tsRNAs (*n* = 243) in Figure 3B. The variation in tsRNAs expression between the two groups of samples is shown by the scatter plot (Figure 3C). Tables 2, 3 summarized the significantly differentially expressed tsRNAs in PAH vs. healthy controls and individually sorted by upregulated and downregulated fold change. For the top 15 upregulated tsRNAs in PAH, the fold change ranged from 29.85- to 3.67-fold, while that of 81.9- to 21.25-fold for the top 15 downregulated tsRNAs.

**TABLE 2:** The top 15 differentially expressed tsRNAs of PAH compared with healthy controls.

Names	Type	Fold change	Length	tsRNA-sequence	*p*-value	Mintbase ID
**5’tiRNA-31-Glu-CTC-16**	5’tiRNA	29.85	31	GTG​GAT​AGC​CCA​GCG​GCT​ATG​GGA​GCC​GGG​C	0.0022	tRF-31-72V1JHQQR03KD
**tRF3b-Tyr-GTA-4**	3’tRF	18.25	22	TCG​ATT​CCA​GCT​CGA​AGG​ACC​A	0.0051	tRF-22-WE8P4U1D2
i-tRF-16:44-Glu-CTC-1	i-tRF	14.59	29	TGG​TTA​GGA​TTC​GGC​GCT​CTC​ACC​GCC​GC	0.0037	tRF-29-9N1EWJQ72S1M
tRF3-23-AspGTC-3	3’tRF	6.66	23	GTT​CGA​TTC​CCC​GAC​GGG​GAG​CC	0.0039	tRF-23-7SIR3DR2DV
**i-tRF-31:54-Val-CAC-1**	i-tRF	6.59	24	CCT​CAC​ACG​CGA​AAG​GTC​CCC​GGT	0.0022	tRF-24-362VO0SR1Z
tRF3a-Asp-GTC-6	3’tRF	6.26	18	TTCCCTGATGGGGAGCCA	0.005	tRF-18-YRX4R0D2
**tRF3a-Asp-GTC-9**	3’tRF	5.92	18	TTCCCTGACGGGGAGCCA	0.003	tRF-18-YRXHR0D2
tRF3-16-Gln-TTG-3	3’tRF	5.38	16	CTCGGTGGGACCTCCA	0.0018	tRF-16-48923RB
tRF5-20-Met-CAT-4	5’tRF	5.21	20	GCC​CTC​TTA​GCG​CAG​CGG​GC	0.0008	tRF-20-P7NPLPMJ
tRF3b-Asp-GTC-4	3’tRF	5.16	22	TCG​ATT​CCC​CAA​CGG​GGA​GCC​A	0.0045	tRF-22-WE8RB86J2
3’tiRNA-40-AspGTC-5	3’tiRNA	4.95	40	CAC​GCG​GGA​GAC​TGG​GGT​TCG​ATT​CCC​TGA​GGA​GGA​GCC​A	0.0034	tRF-40-2VR0E8R9593U6KF6
tRF5-26-Arg-TCT-4	5’tRF	4.30	26	GTC​TCT​GTG​GCG​CAA​TGG​ACG​AGC​GC	0.0011	tRF-26-S998LO9D5HD
i-tRF-38:54-Gly-CCC-2	i-tRF	4.21	16	TTCTTGCGACCCGGGT	0.0026	tRF-16-YZQD3KE
i-tRF-16:54-Gly-CCC-2	i-tRF	4.02	37	TGG​TAT​CAT​GCA​AGA​TTC​CCA​TTC​TTG​CGA​CCC​GGG​T	0.0009	tRF-37-9L8422YRI7XUK8N
i-tRF-35:54-Gly-CCC-2	i-tRF	3.67	19	CCA​TTC​TTG​CGA​CCC​GGG​T	0.0010	tRF-19-KNNVORIZ

Bold font are the candidate tsRNAs selected for bioinformatics and real-time PCR.

**TABLE 3:** The top 15 differentially expressed downregulated tsRNAs of PAH compared with healthy controls.

Names	Type	Fold change	Length	tsRNA-sequence	*p*-value	Mintbase ID
i-tRF-15:32-Val-AAC-2	i-tRF	0.01221	18	GTGGTCATCACGTTCGCC	0.00041	tRF-18-73J6M9DV
**i-tRF-8:32-Val-AAC-2**	i-tRF	0.01333	25	TAG​TGT​AGT​GGT​CAT​CAC​GTT​CGC​C	0.00036	tRF-25-V4V4SQ2WW1
i-tRF-8:32-Val-AAC-1	i-tRF	0.01445	25	TAG​TGT​AGT​GGT​TAT​CAC​GTT​CGC​C	0.00455	tRF-25-V4V47Q2WW1
i-tRF-7:32-Val-AAC-1	i-tRF	0.02488	26	GTA​GTG​TAG​TGG​TTA​TCA​CGT​TCG​CC	0.00566	tRF-26-S3S3RX8HYVD
**i-tRF-2:30-His-GTG-1**	i-tRF	0.02641	29	CCG​TGA​TCG​TAT​AGT​GGT​TAG​TAC​TCT​GC	0.00002	tRF-29-34HWH3RXSIHM
5’tiRNA-34-ArgTCG-1	5’tiRNA	0.02927	34	GGC​CGC​GTG​GCC​TAA​TGG​ATA​AGG​CGT​CTG​ACT​T	0.00001	tRF-34-6SM83O9EDJSYEQ
**5’tiRNA-33-Lys-TTT-4**	5’tiRNA	0.03001	32	GCC​TGG​ATA​GCT​CAG​TCG​GTA​GAG​CAT​CAG​ACT	0.00042	tRF-33-PY5P4PW3FJHPW
i-tRF-23:41-Gln-TTG-1	i-tRF	0.03209	19	CAC​TCT​GGA​CTT​TGA​ATC​C	0.00001	tRF-19-299DZUFJ
5’tiRNA-35-Arg-TCT-1	5’tiRNA	0.03520	35	GGC​TCC​GTG​GCG​CAA​TGG​ATA​GCG​CAT​TGG​ACT​TC	0.00186	tRF-35-69M8LO9EFVI8E9
5’tiRNA-35-Ala-AGC-1	5’tiRNA	0.03930	35	GGG​GGT​ATA​GCT​CAG​TGG​TAG​AGC​GCG​TGC​TTA​GC	0.00024	tRF-35-RKVP4P9L5HMVYJ

Bold font are the candidate tsRNAs selected for bioinformatics and real-time PCR.

### Validation with real-time PCR

**FIGURE 4:**
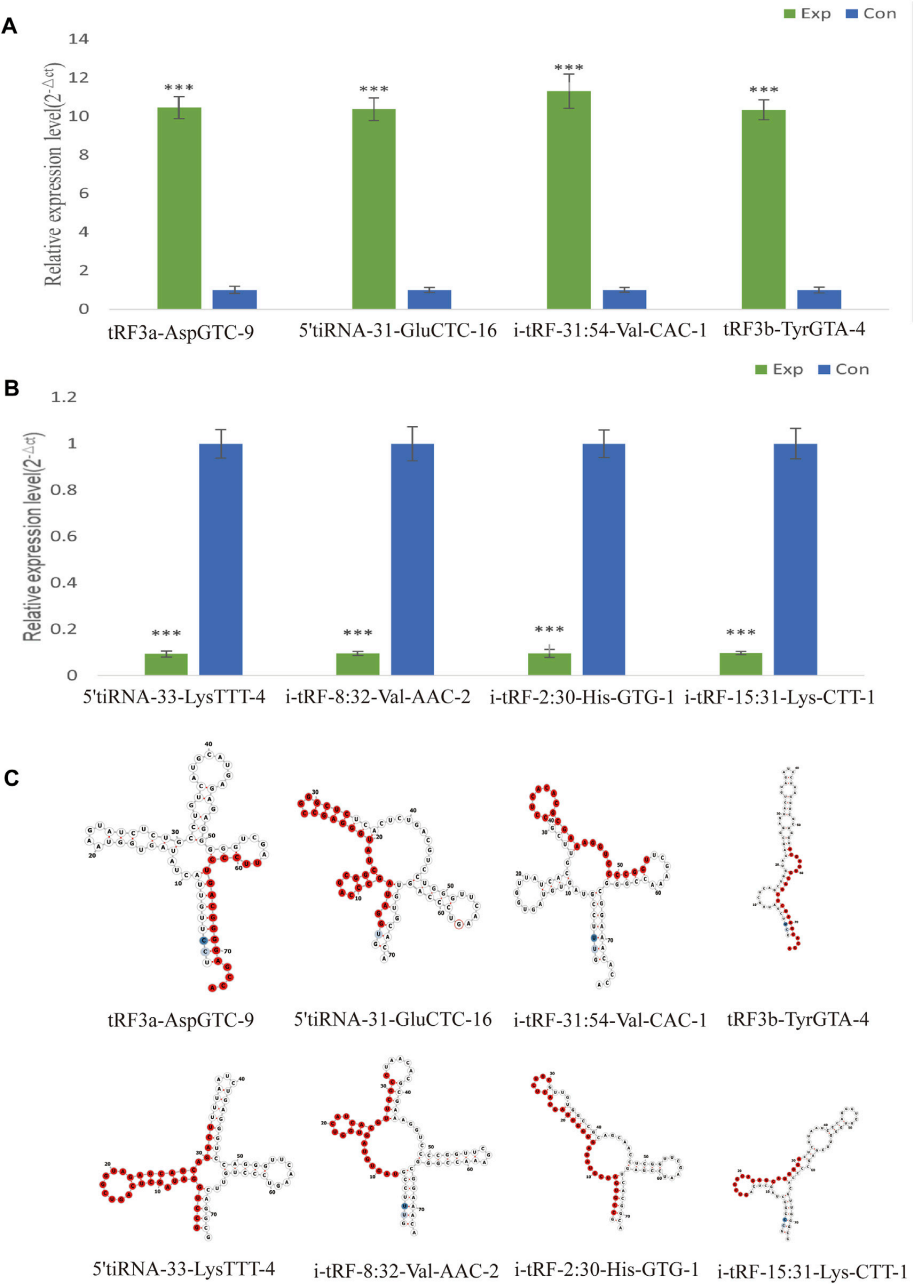
Verification of the expression of significantly dysregulated tsRNAs by real time-PCR. We selected 8 tsRNAs dysregulated in PAH for custom real time-PCR. **(A)** Expression of 4 upregulated tsRNAs in PAH. **(B)** Expression of 4 downregulated tsRNAs in PAH. ***p < 0.0001 as indicated. **(C)** Examples of tRNA or pre-tRNA structures of the eight validated tsRNAs were depicted. The red nucleotides indicate the presence of the eight validated tsRNAs sequences. Exp, PAH patients; Con, healthy controls.

Selecting tsRNAs standard included a higher fold change, lower q-value, and higher CMP. After the integrated data were unified, 8 differentially expressed tsRNAs were screened to verify the microarray results, four of which were remarkably upregulated, and the others were downregulated. As a result, compared with the healthy control, tRF3a-AspGTC-9 (10.46fold), 5’tiRNA-31-GluCTC-16 (10.38fold), i-tRF-31:54-Val-CAC-1 (11.31-fold) and tRF3b-TyrGTA-4 (10.35fold) were all statistically upregulated in PAH group, and 5’tiRNA-33-LysTTT-4 (0.09-fold), i-tRF-8:32-Val-AAC-2 (0.10-fold), i-tRF-2:30-His-GTG-1 (0.10-fold), and i-tRF-15:31-Lys-CTT-1 (0.10-fold) were all significantly downregulated in the PAH group, which is consistent with the small RNA microarray (Figures 4A, B; Supplementary Table S3). The identified tsRNAs were determined as 3’tRF, 5’tRF, i-tRF, and 5’tiRNA, respectively, based on sites of cleavage on the tRNAs’ cloverleaf secondary structure (Figure 4C).

### Potential mRNA of validated tsRNA

**FIGURE 5:**
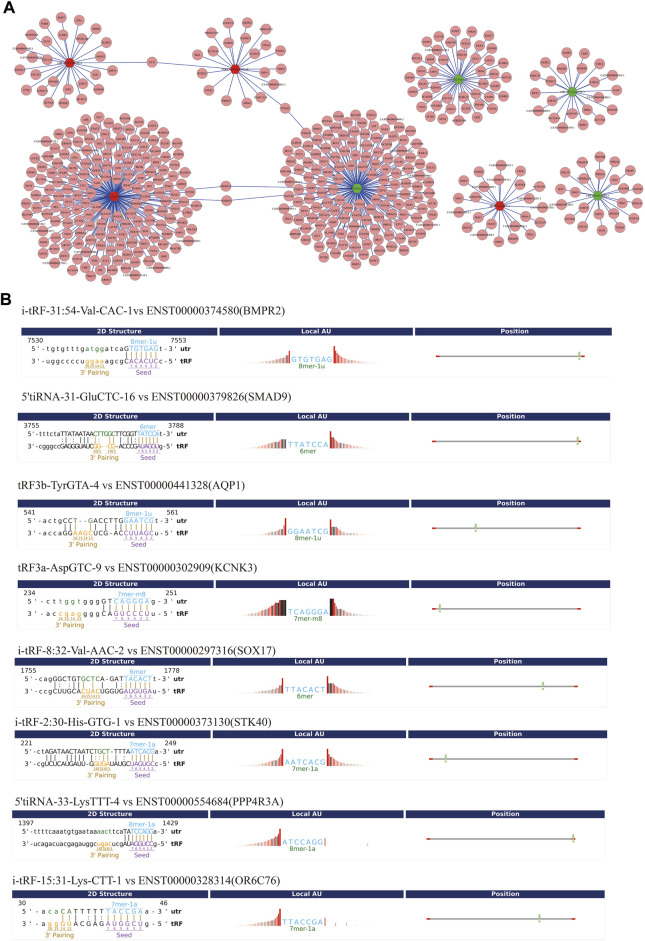
Gene ontology (GO) enrichment analyses for the eight validated tsRNAs. **(A)** Putative target mRNA genes of the eight validated tsRNAs. **(B)** The binding region and seed sequence of the tsRNAs and mRNAs (BMPR2, SMAD9, AQP1, KCNK3, SOX17, STK40, PPP4R3A, and OR6C76).

To improve prediction reliability, targetScan and miRanda were used to construct the PAH-related tsRNA-target gene network. We have projected the potential target genes of verified tsRNAs (tRF3a-Asp-GTC-9, 5’tiRNA-31-Glu-CTC-16, i-tRF-31:54-Val-CAC-1, tRF3b-Tyr-GTA-4, 5’tiRNA-33-Lys-TTT-4, i-tRF-8:32-Val-AAC-2, i-tRF-2:30-His-GTG-1, and i-tRF-15:31-Lys-CTT-1). Finally, we obtained 174, 21, 27, 23, 152, 44, 23, and 22 potential target genes, respectively and revealed the genes with context score < −0.4 (Figure 5A). Recent studies have shown that tsRNAs identify target mRNAs and compete for binding mRNAs during transcription, replacing the untranslated region in mRNAs and reducing the stability of transcripts, thus inhibiting mRNA expression. Figure 5B shows the binding sites and seed sequences of the eight tsRNAs as well as their target mRNAs. Eight mRNA genes [bone-forming protein type II receptor (BMPR2), SMAD family member 9 (SMAD9), potassium two pore domain channel subfamily K member 3 (KCNK3), aquaporin1 (AQP1), sex-determining region Y-box 17 (SOX17), serine/threonine kinase 40 (STK40), protein phosphatase 4 regulatory subunit 3A (PPP4R3A), olfactory receptor family 6 subfamily C member 76 (OR6C76)], were individually the target genes of the validated tsRNAs.

### GO analysis

**FIGURE 6:**
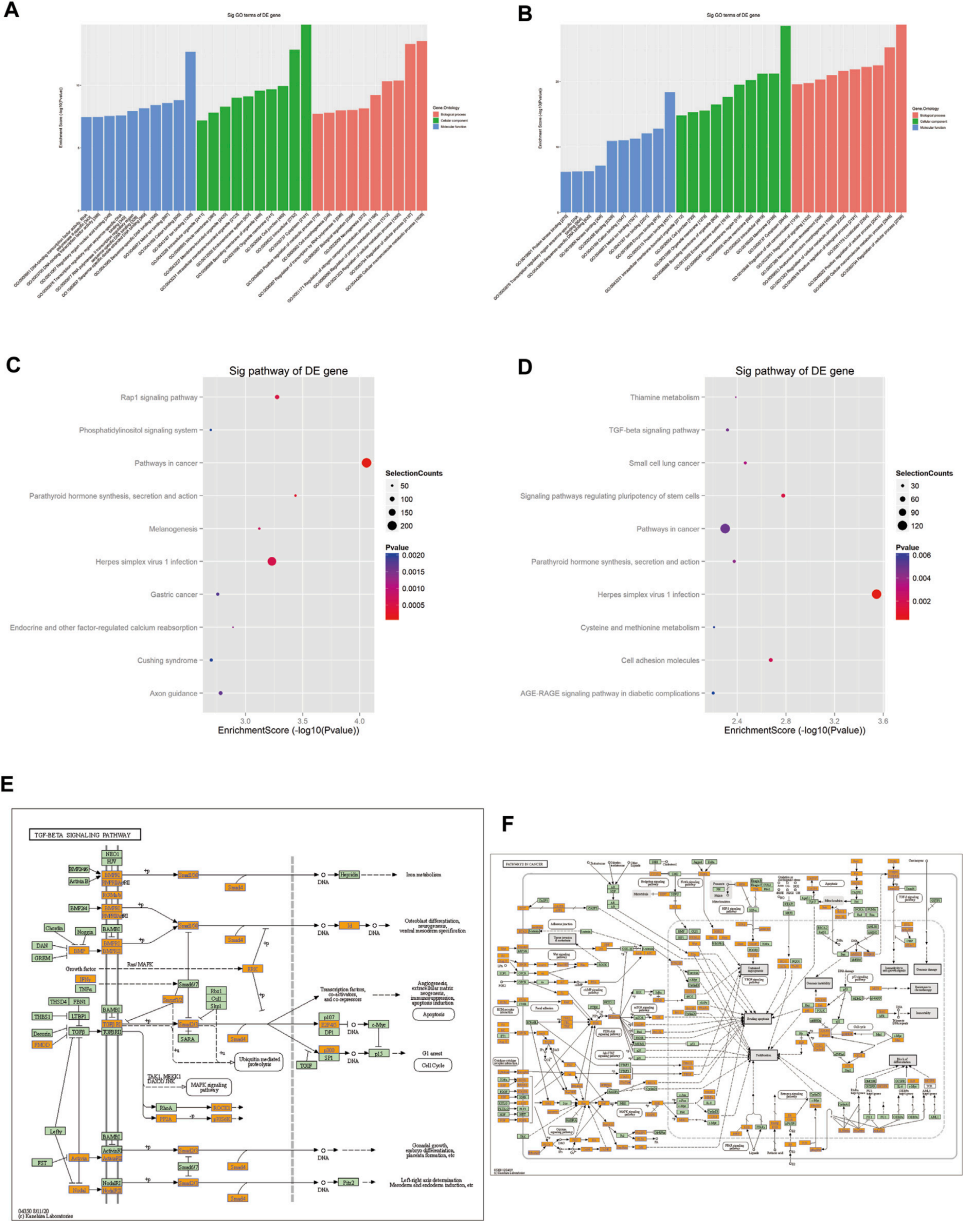
Prediction of signaling pathways of the eight validated tsRNAs. Analysis showed that the target genes of **(A)** the four upregulated tsRNAs and **(B)** the four downregulated tsRNAs’ biological function. KEGG analyses of pathways on upregulated tsRNAs **(C)** and downregulated tsRNAs **(D)**. **(E)** Mapping of the TGF-β signaling pathway in upregulated tsRNAs. **(F)** Mapping of the cancer signaling pathway in downregulated tsRNAs. Yellow marked nodes are associated with downregulated genes, orange marked nodes are associated with upregulated or only whole dataset genes, green nodes have no significance.

GO analysis revealed that the target genes of the selected tsRNAs were most abundant in certain biological processes, molecular functions, and cellular components, as shown in Figures 6A, B (Supplementary Table S4). The potential target genes of the four upregulated tsRNAs are mainly situated in the cytoplasm, intracellular, and cell junction, involved in cellular macromolecule metabolic process, regulation of cellular process, and regulation of cellular metabolic process. Their major molecular functions are ion binding, cation binding, and metal ion binding. For the four downregulated tsRNAs, the potential target genes are mainly located in cytoplasm, membrane, and intracellular, participating regulation of cellular process, cellular macromolecule metabolic process, and positive regulation of the cellular process. Their major molecular functions are protein binding, enzyme binding, and ion binding. In conclusion, these data suggest that target genes of differentially expressed tsRNAs may be engaged in cell metabolism, proliferation, differentiation, and apoptosis.

### KEGG analysis

The analysis implied that the eight tsRNAs are involved in 89 KEGG pathways that corresponded to the target genes related to the tsRNAs (*p* < 0.05). Figures 6C, D present a dot matrix depicting the log10 *p*-values of KEGG pathways. Log10 (*p*-value) represents an enrichment score of path-related importance. Out of them, the four upregulated tsRNAs play a key role in TGF-β pathways (Figure 6E) and the four downregulated tsRNAs play an important role in pathways in cancer (Figure 6F). Among these pathways, peroxisome proliferator-activated receptor (PPAR) signaling, BMPR2 signaling, and HIF-1 (hypoxia-inducible factor-1) signaling have been demonstrated to be associated with PAH. These preliminary findings underscore the disruptive implications of tsRNAs in PAH and allow for more profound research that is still needed to confirm their role in the pathogenesis of PAH and to explore new non-invasive biomarkers.

## Discussion

Pulmonary arterial hypertension is an idiopathic cardiopulmonary disease characterized by excessive vascular cell proliferation and resistance to apoptosis, as well as inflammation, thrombosis, and vasoconstriction, which leads to obstruction of small pulmonary arteries. Previous studies have demonstrated that genetic factors served a pivotal role in PAH development and progression (Zhu et al., 2019). As reported in previous studies, tsRNAs can precisely regulate gene expression (Ivanov et al., 2011; Gebetsberger et al., 2017). Through small RNA microarray, we found that 816 tsRNAs in the idiopathic PAH vs. healthy controls were different in expression and may be related to the pathogenesis of PAH. Among the ratio of subtype tsRNAs, 5’tRF was the main type of dysregulated tsRNAs in the PAH group. Whether the dysfunction of 5’tRF in PAH can regulate the gene expression, which leads to PAH and promote the disease development, is still worthy of further study. The subtype tsRNAs against tRNA iso-decoders are comprehensive Cys-GCA, Gly-GCC, Gly-TCC, His-GTG, Ile-AAT, iMet-CAT, Tyr-GTA, and Val-TAC in the two groups. Additionally, the bioinformatics analysis implied that the tsRNAs could target key PAH-related genes such as BMPR2, which is involved in signaling pathways that regulate cell growth and differentiation and in which mutations are the most common genetic cause of PAH (Happé et al., 2020).

In following studies, eight significantly differentially expressed tsRNAs were selected for validation. Four upregulated tsRNAs, namely, i-tRF-31:54-Val-CAC-1, 5’tiRNA-31-Glu-CTC-16, tRF3a-AspGTC-9, and tRF3b-TyrGTA-4, and four downregulated tsRNAs, namely, 5’tiRNA-33-LysTTT-4, i-tRF-8:32-Val-AAC-2, i-tRF-2:30-His-GTG-1, and i-tRF-15:31-Lys-CTT-1 were identified as consistent with the microarray results. It is still essential to enlarge the specimens while testing the levels of tsRNAs in plasma to confirm their stability as biomarkers in the future.

It has been previously shown that miRNAs and lncRNAs are differentially expressed in PAH patients, and both are thought to be involved in the pathogenesis of PAH (Deng et al., 2015; Cai et al., 2018; Omura et al., 2020; Sindi et al., 2020; Xu et al., 2021). miRNAs such as miR-29b, miR-140-5p, and miR-21 have been identified in different PAH rat models and human tissues, and dysregulated miRNAs lead to excessive proliferation and apoptosis-resistant phenotypes of pulmonary arterial smooth muscle cells (PASMCs) (Rothman et al., 2016; Caruso et al., 2017; Ng et al., 2018). What’s more, mutations in BMPR2 were shown to be associated with miR-145 positively correlated (Caruso et al., 2012). miR-21 dysregulated expression in Hypoxia-induced PASMCs regulates several downstream genes, including programmed cell death protein 4, Sprouty 2, and peroxisome proliferator-activated receptor (Wuttge et al., 2021). Several studies have shown that tsRNAs, the newest non-coding RNA, can affect mRNA translation by directly regulating mRNAs in a manner similar to miRNAs. But tsRNA targets are not only located in the 3’-untranslated region of mRNA but also in the 5’ -untranslated region or coding sequence. More interestingly, tsRNAs can preferentially target mRNAs that inhibit key components of the translation machinery, such as ribosomal proteins and translation initiation or elongation factors, thereby inhibiting overall protein translation (Luo et al., 2018).

To predict the function of validated tsRNAs, two databases, miRanda and TargetScan, were used to estimate the intersection of the target genes, which included BMPR2 and AQP1. These differentially expressed tsRNAs were found to be abundant in some important biological processes (i.e., regulation of cellular metabolic process), molecular functions (i.e., ion binding), and cellular components (i.e., organelle membrane) for PAH. Some signaling pathways such as the pathways in BMPR2, PPAR, and the HIF-1 signaling pathway were also tightly related to PAH. These pathways are associated with the formation of abnormal proliferation and apoptosis resistance of PASMCs, which are important processes in PAH. Previous studies have reported that BMPR2 gene mutation in PAH is significantly downregulated, and the present study revealed that BMPR2 was targeted by i-tRF-31:54-Val-CAC-1. BMPR2 is a receptor protein for the bone-forming protein, a member of the transforming growth factor receptor superfamily. Dysfunctional BMPR2 signaling is a key feature of PAH, and 70%–80% of heritable PAH and 10%–20% of PAH are associated with BMPR2 variants. The BMPR2 mutation is associated with abnormal mitochondrial function and insulin metabolism in cardiac myocytes, with enhanced insulin resistance, reduced glucose uptake, and enhanced lipid uptake, resulting in changes in right ventricular lipotoxicity in PAH. In conclusion, our results suggest that the function of tsRNA in PAH may engage in the regulation of key mRNAs and reveal that increased expression of i-tRF-31:54-Val-CAC-1 contributes to the development of PAH via targeting BMPR2. In addition, AQP1 is an important factor in the progression of PAH and tRF3b-TyrGTA-4 may be involved in the progression of PAH by targeting AQP1. Such the pathological basis as migration and proliferation of PASMCs are mainly required for pulmonary artery remodeling and thus PAH. AQP1 contributes a lot to promoting the migration of PASMCs and endothelium. It has been shown that hypoxia promotes AQP1 expression in pulmonary arteries and that AQP1 regulates the proliferation and migration of PASMCs through linked proteins (Gräf et al., 2018; Yun et al., 2021). Elevated AQP1 levels promote PASMC proliferation and migration by upregulating linked proteins, leading to the expression of MYC proto-oncogene protein and cyclinD1 (Yun et al., 2017). It’s reported that tsRNA has a direct inhibitory action on protein synthesis by post-transcriptional regulation of gene expression via directly inhibiting protein synthesis (Bąkowska-Żywicka et al., 2016) or serve as a genetic participant in the modulation of DNA damage (Maute et al., 2013). Intriguingly, tsRNAs could be linked with AGO proteins, and then the target mRNA could be identified by the AGO complexes (Kuscu et al., 2018). AGO proteins in humans play their post-transcriptional regulatory roles by forming effector complexes with four members (AGO1-4) expressed in cells and tissues and associated with different miRNAs and tsRNAs (Kuscu et al., 2018). It’s speculated by us that the dysregulated expression of tsRNA may serve as a regulator in form of AGO-bound or function similarly to miRNA in the RNA biosynthetic process and DNA damage at the onset of PAH or during the disease advancement. Further studies would pay much more attention to its potential biological functional acting as a miRNA in the disease.

There are several limitations of the research. First, only expression profiles, a small sample size validation, and the functional predictions are completed in this research; determining the roles of the tsRNAs *in vitro* and *in vivo* is expected to be investigated in the next step; the current findings are required to validate by clinical samples and larger sample sizes in future studies. Second, since all the samples in this study were from blood, the available results do not indicate whether tsRNAs originate from lung tissue and how it affects pulmonary vascular disease. We will establish animal models of PAH to be explored in the next studies. Furthermore, target prediction using miRNA pattern-like methods to predict tsRNAs is relatively limited. Hence, it is imperative to establish more accurate prediction methods to better identify and understand the functions of tsRNAs. The particular interactions and sites of binding between mRNAs and tsRNAs are needed to explore. Nevertheless, this is the first study to research tsRNAs in PAH and we have used samples of idiopathic PAH.

## Conclusion

This study investigated tsRNA profiles in PAH and found that the expression of several dysregulated tsRNAs may be closely related to the pathogenesis and advancement of PAH. Our study is promising to provide further insights into the pathogenesis of diseases and likely fuel the discovery of new therapies.

## Data Availability

The datasets presented in this study can be found in online repositories. The names of the repository/repositories and accession number(s) can be found below: https://www.ncbi.nlm.nih.gov/geo/query/acc.cgi?acc=GSE228750.

## Supplementary material

SUPPLEMENTARY FIGURE S1 Study flow chart.
