# Supplementary material for: Differential expression spectrum and targeted gene prediction of tRNA-derived small RNAs in idiopathic pulmonary arterial hypertension
Source: Front Mol Biosci. 2023 Jul 11;10:1204740. doi: 10.3389/fmolb.2023.1204740 (PMC10367008; doi:10.3389/fmolb.2023.1204740)
Supplement: Supplementary file 1 [file Table1.DOCX]

Supplementary Table S1. Primers for Real-time PCR.

| Gene | Primers | Annealing temperature (℃) | Length (bp) |
| --- | --- | --- | --- |
| U6 | F:5’GCTTCGGCAGCACATATACTAAAAT3’  R:5’CGCTTCACGAATTTGCGTGTCAT3’ | 60 | 89 |
| i-tRF-31:54-Val-CAC-1 | F:5’ TACAGTCCGACGATCCCTCAC3’  R:5’ CGATCTACCGGGGACCTTTC3’ | 60 | 45 |
| 5'tiRNA-31-GluCTC-16 | F:5’ AGTCCGACGATCGTGGATAGC3’  R:5’ GATCTGCCCGGCTCCCATA3’ | 60 | 48 |
| tRF3a-AspGTC-9 | F:5’ CTACAGTCCGACGATCTTCCCT3’  R:5’ TGCTCTTCCGATCTTGGCTC3’ | 60 | 48 |
| tRF3b-TyrGTA-4 | F:5’ AGTCCGACGATCTCGATTCC3’  R:5’ CTCTTCCGATCTTGGTCCTTC3’ | 60 | 46 |
| i-tRF-8:32-Val-AAC-2 | F:5’ GTCCGACGATCTAGTGTAGTGGTC3’  R:5’ CTTCCGATCTGGCGAACGT3’ | 60 | 46 |
| i-tRF-2:30-His-GTG-1 | F:5’ GATCCCGTGATCGTATAGTGG3’  R:5’ GCTCTTCCGATCTGCAGAGT3’ | 60 | 46 |
| i-tRF-15:31-Lys-CTT-1 | F:5’ AGTCCGACGATCGTCGGTAG3’  R:5’ GTGCTCTTCCGATCTTCCCA3’ | 60 | 44 |
| 5'tiRNA-33-LysTTT-4 | F:5’ ACGATCGCCTGGATAGCTCA3’  R:5’ TCTTCCGATCTAGTCTGATGCTCT3’ | 60 | 50 |

U6 was used as the internal control. F, forward; R, reverse.

Supplementary Table S2 RNA quantification and quality assurance by spectrophotometry.

| Group | OD260/280 ratio | Concentration (ng/µl) | Quantity (ng) | Result |
| --- | --- | --- | --- | --- |
| PAH | 1.50 | 60.28 | 904.15 | Pass |
| Controls | 1.53 | 60.90 | 913.45 | pass |

PAH: pulmonary arterial hypertension; OD260/280, ratio of optical density at 260 and 280 nm;

Supplementary Table S3 Comparison of real-time PCR data with small RNA microarray data for tsRNAs.

| PAH/control ratio | Method | |
| --- | --- | --- |
|  | real-time PCR | small RNA microarray |
| tRF3a-AspGTC-9 | 10.46 | 5.92 |
| 5'tiRNA-31-GluCTC-16 | 10.38 | 29.85 |
| i-tRF-31:54-Val-CAC-1 | 11.31 | 6.59 |
| tRF3b-TyrGTA-4 | 10.35 | 18.25 |
| 5'tiRNA-33-LysTTT-4 | 0.09 | 0.048 |
| i-tRF-8:32-Val-AAC-2 | 0.10 | 0.013 |
| i-tRF-2:30-His-GTG-1 | 0.10 | 0.026 |
| i-tRF-15:31-Lys-CTT-1 | 0.10 | 0.048 |

Real-time PCR: real-time polymerase chain reaction.

Supplementary Table S4 The significant enriched GO analysis of target genes.

| ID | Pathway | Type | Count | *P*-value |
| --- | --- | --- | --- | --- |
| Upregulated tsRNAs | | |  |  |
| GO:0044260 | cellular macromolecule metabolic process | Biological process | 1628 | 2.9378E-14 |
| GO:0050794 | regulation of cellular process | Biological process | 2137 | 5.026E-14 |
| GO:0031323 | regulation of cellular metabolic process | Biological process | 1250 | 4.2118E-11 |
| GO:0080090 | regulation of primary metabolic process | Biological process | 1212 | 4.7221E-11 |
| GO:0051171 | regulation of nitrogen compound metabolic process | Biological process | 1168 | 6.0163E-10 |
| GO:0022008 | neurogenesis | Biological process | 372 | 7.0375E-09 |
| GO:0065007 | biological regulation | Biological process | 2356 | 9.2542E-09 |
| GO:0006357 | regulation of transcription by RNA polymerase II | Biological process | 536 | 9.8546E-09 |
| GO:0000902 | cell morphogenesis | Biological process | 246 | 1.5775E-08 |
| GO:0009893 | positive regulation of metabolic process | Biological process | 779 | 1.8744E-08 |
| GO:0005737 | cytoplasm | Cellular component | 2191 | 1.4697E-15 |
| GO:0005622 | intracellular | Cellular component | 2752 | 1.4436E-13 |
| GO:0030054 | cell junction | Cellular component | 465 | 1.1143E-10 |
| GO:0031090 | organelle membrane | Cellular component | 741 | 2.0802E-10 |
| GO:0098588 | bounding membrane of organelle | Cellular component | 468 | 2.696E-10 |
| GO:0012505 | endomembrane system | Cellular component | 920 | 7.568E-10 |
| GO:0043231 | intracellular membrane-bounded organelle | Cellular component | 2123 | 9.6791E-10 |
| GO:0043227 | membrane-bounded organelle | Cellular component | 2420 | 5.1668E-09 |
| GO:0098805 | whole membrane | Cellular component | 380 | 1.5557E-08 |
| GO:0043229 | intracellular organelle | Cellular component | 2411 | 6.3811E-08 |
| GO:0043167 | ion binding | Molecular function | 1305 | 2.0812E-13 |
| GO:0043169 | cation binding | Molecular function | 905 | 1.5597E-09 |
| GO:0046872 | metal ion binding | Molecular function | 887 | 2.6103E-09 |
| GO:0043565 | sequence-specific DNA binding | Molecular function | 395 | 3.8245E-09 |
| GO:1990837 | sequence-specific double-stranded DNA binding | Molecular function | 369 | 6.8552E-09 |
| GO:0000977 | RNA polymerase II transcription regulatory region sequence-specific DNA binding | Molecular function | 328 | 1.1625E-08 |
| GO:0000976 | transcription regulatory region sequence-specific DNA binding | Molecular function | 345 | 2.6065E-08 |
| GO:0001067 | regulatory region nucleic acid binding | Molecular function | 345 | 2.8111E-08 |
| GO:0003700 | DNA-binding transcription factor activity | Molecular function | 356 | 3.3988E-08 |
| GO:0000981 | DNA-binding transcription factor activity, RNA polymerase II-specific | Molecular function | 343 | 3.4928E-08 |
| Downregulated tsRNA | | | | |
| GO:0050794 | regulation of cellular process | Biological process | 3766 | 2.0285E-29 |
| GO:0044260 | cellular macromolecule metabolic process | Biological process | 2846 | 5.8746E-26 |
| GO:0048522 | positive regulation of cellular process | Biological process | 2001 | 3.3021E-23 |
| GO:0051179 | localization | Biological process | 2354 | 5.9146E-23 |
| GO:0048518 | positive regulation of biological process | Biological process | 2191 | 1.3875E-22 |
| GO:0031323 | regulation of cellular metabolic process | Biological process | 2202 | 2.4161E-22 |
| GO:0009653 | anatomical structure morphogenesis | Biological process | 1059 | 1.0602E-21 |
| GO:0007399 | nervous system development | Biological process | 935 | 4.865E-21 |
| GO:0023051 | regulation of signaling | Biological process | 1332 | 1.7077E-20 |
| GO:0010646 | regulation of cell communication | Biological process | 1318 | 2.5435E-20 |
| GO:0005737 | cytoplasm | Cellular component | 3849 | 3.0175E-29 |
| GO:0016020 | membrane | Cellular component | 3186 | 5.933E-22 |
| GO:0005622 | intracellular | Cellular component | 4819 | 6.074E-22 |
| GO:0098805 | whole membrane | Cellular component | 692 | 6.0444E-21 |
| GO:0098590 | plasma membrane region | Cellular component | 519 | 3.0611E-20 |
| GO:0012505 | endomembrane system | Cellular component | 1616 | 2.2456E-18 |
| GO:0098588 | bounding membrane of organelle | Cellular component | 809 | 3.1804E-17 |
| GO:0031090 | organelle membrane | Cellular component | 1279 | 2.6648E-16 |
| GO:0030054 | cell junction | Cellular component | 793 | 4.6157E-16 |
| GO:0043231 | intracellular membrane-bounded organelle | Cellular component | 3712 | 1.4806E-15 |
| GO:0005515 | protein binding | Molecular function | 4577 | 4.1344E-19 |
| GO:0019899 | enzyme binding | Molecular function | 875 | 1.5205E-13 |
| GO:0043167 | ion binding | Molecular function | 2201 | 8.1319E-13 |
| GO:0046872 | metal ion binding | Molecular function | 1521 | 5.4431E-12 |
| GO:0043169 | cation binding | Molecular function | 1547 | 9.3844E-12 |
| GO:0005488 | binding | Molecular function | 5328 | 1.1898E-11 |
| GO:0019900 | kinase binding | Molecular function | 306 | 7.0142E-08 |
| GO:0043565 | sequence-specific DNA binding | Molecular function | 630 | 5.1769E-07 |
| GO:0000976 | transcription regulatory region sequence-specific DNA binding | Molecular function | 554 | 5.5978E-07 |
| GO:0019901 | protein kinase binding | Molecular function | 270 | 6.0978E-07 |
