# Supplementary figures and images for: Differential expression spectrum and targeted gene prediction of tRNA-derived small RNAs in idiopathic pulmonary arterial hypertension
Source: Front Mol Biosci. 2023 Jul 11;10:1204740. doi: 10.3389/fmolb.2023.1204740 (PMC10367008; doi:10.3389/fmolb.2023.1204740)

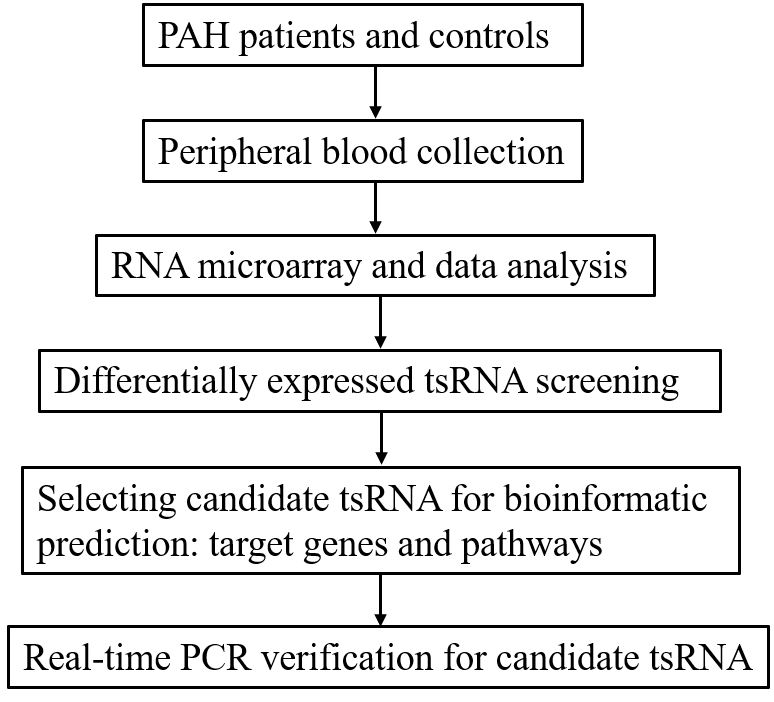

Supplement: Supplementary file 2 [file Image1.tif]
